# A comparison of the 33-item Hypomania Checklist with the 33-item Hypomania Checklist-external assessment for the detection of bipolar disorder in adolescents

**DOI:** 10.1186/s40345-021-00246-0

**Published:** 2021-12-18

**Authors:** Xu Chen, Wei Bai, Na Zhao, Sha Sha, Teris Cheung, Gabor S. Ungvari, Yuan Feng, Yu-Tao Xiang, Jules Angst

**Affiliations:** 1 The National Clinical Research Center for Mental Disorders & Beijing Key Laboratory of Mental Disorders, Beijing Anding Hospital & the Advanced Innovation Center for Human Brain Protection, Capital Medical University, School of Mental Health, Xicheng District, Beijing, 100088 China; 2 Unit of Psychiatry, Department of Public Health and Medicinal Administration, & Institute of Translational Medicine, Faculty of Health Sciences, University of Macau, Macao SAR, China; 3 Centre for Cognitive and Brain Sciences, University of Macau, Macao SAR, China; 4 Institute of Advanced Studies in Humanities and Social Sciences, University of Macau, Macao SAR, China; 5 Center for Cognition and Brain Disorders, Institutes of Psychological Sciences, Hangzhou Normal University, Hangzhou, China; 6 School of Nursing, Hong Kong Polytechnic University, Hong Kong SAR, China; 7 Division of Psychiatry, School of Medicine, University of Western Australia, Perth, Australia; 8 Section of Psychiatry, University of Notre Dame Australia, Fremantle, Australia; 9 Zurich University Psychiatric Hospital, Lenggstrasse 31, P.O. Box 8032, Zurich, Switzerland

**Keywords:** Adolescents, Bipolar disorder, HCL-33, Self-assessment, External assessment

## Abstract

**Background:**

Adolescents with bipolar disorder (BD) are often misdiagnosed as having major depressive disorder (MDD), which delays appropriate treatment and leads to adverse outcomes. The aim of this study was to compare the performance of the 33-item Hypomania Checklist (HCL-33) with the 33-item Hypomania Checklist- external assessment (HCL-33-EA) in adolescents with BD or MDD.

**Methods:**

147 adolescents with BD and 113 adolescents with MDD were consecutively recruited. The HCL-33 and HCL-33-EA were completed by patients and their carers, respectively. The sensitivity, positive predictive value (PPV), specificity, negative predictive value (NPV), and area under the curve (AUC) were calculated and compared between the two instruments, using cut-off values based on the Youden’s index.

**Results:**

The total scores of the HCL-33 and HCL-33-EA were positively and significantly correlated (*rs* = 0.309, *P* < 0.001). Compared to the HCL-33, the HCL-33-EA had higher sensitivity and NPV (HCL-33: sensitivity = 0.58, NPV = 0.53; HCL-33-EA: sensitivity = 0.81, NPV = 0.60), while the HCL-33 had higher specificity and PPV (HCL-33: specificity = 0.61, PPV = 0.66; HCL-33-EA: specificity = 0.37, PPV = 0.63).

**Conclusion:**

Both the HCL-33 and HCL-33-EA seem to be useful for screening depressed adolescents for BD. The HCL-33-EA would be more appropriate for distinguishing BD from MDD in adolescents due to its high sensitivity in Chinese clinical settings.

## Introduction

Bipolar Disorder (BD) is a chronic mood disorder characterized by depressive and manic or hypomanic episodes (Phillips and Kupfer 2013). Compared to manic episodes, bipolar depressive episodes usually have higher rates of morbidity and mortality (McIntyre and Calabrese 2019). A major challenge in clinical practice is to diagnose BD accurately, as it is difficult to differentiate from other psychiatric disorders, in particular major depressive disorder (MDD). The difficulty arises because the first episode of mood disturbance in BD is frequently depression (McIntyre and Calabrese 2019). In addition, patients are more likely to seek medical treatment for their depressive symptoms than when they are experiencing manic and/hypomanic symptoms (Cuomo et al. 2020; Zimmerman and Galione 2011). The misdiagnosis of BD as MDD may have serious clinical consequences (Patella et al. 2019; Hwang et al. 2010) due to the delay of appropriate treatment and the inappropriate prescription of antidepressants that increase the risk of chronicity and recurrence of BD (McIntyre and Calabrese 2019; Fagiolini et al. 2013).

To improve accuracy in diagnosing BD, standardized and structured or semi-structured diagnostic interviews have been developed, such as the Mini-International Neuropsychiatric Interview (MINI) (Lecrubier et al. 1997), and the Structured Clinical Interview for DSM-5 (SCID-5) (First et al. 2015). These interviews are comprehensive, but time-consuming and their administration is labor-intensive and expensive in clinical or research settings (Nejati et al. 2020). Although comprehensive clinical assessment is essential and irreplaceable, tailor-made screening tools (Hong et al. 2014, Bae et al. 2014) can assist in detecting BD and minimize the risk of misdiagnosis. A study conducted in advanced practice registered nurses reported that screening depressed patients using validated screening tools (e.g., the Mood Disorder Questionnaire (MDQ)) in primary care may reduce the time-lag to the diagnosis and treatment of BD (Kriebel-Gasparro 2016).

The 32-item Hypomania Checklists (HCL-32) is a widely used self-report screening tool for assessing bipolarity in mood disorders. The 33-item Hypomania Checklist (HCL-33) is a modified version of the HCL-32. The Chinese version of the HCL-33 has been validated in both adult (Feng et al. 2016) and adolescent (Zhang et al. 2021) samples to screen for BD in depressed patients. An HCL-33-external assessment version (HCL-33-EA) has been recently developed to rate patients’ symptoms by their carers (e.g., family members, friends, etc.) (Łojko et al. 2016). Given that individuals in a manic or hypomanic state are often unaware of changes in their mood and behavior, carers are privileged observers who can provide valuable additional information to the clinician. The Chinese version of the HCL-33-EA has also been validated in adults (Fang et al. 2019).

To the best of our knowledge, no study has directly compared the properties of the HCL-33 and HCL-33-EA in adolescents. Thus, the aim of this study was to compare the sensitivity, specificity, positive predictive value (PPV), negative predictive value (NPV), and area under the curve (AUC) of the HCL-33 and the HCL-33-EA in Chinese adolescents.

## Method

### Study sample and sites

This study was conducted between August 2020 and November 2020 in the Department of Child Psychiatry at Beijing Anding Hospital of Capital Medical University, a major tertiary psychiatric hospital in China. Participants who met the following inclusion criteria were consecutively recruited during the study period: (1) aged between 13 and 17 years; (2) diagnosed with BD or MDD according to the International Classification of Diseases, Tenth Revision (ICD-10) (WHO 1992) based on a diagnostic interview by two senior psychiatrists; (3) had a current depressive episode defined as a total score of 7 or higher on the 17-item Hamilton Depression Rating Scale (HAMD) (Hamilton 1960; Xie and Shen 1984); (4) were able to understand the aim and contents of the assessment and to provide verbal informed consent, whilst their legal guardians gave written informed consent. Adolescents with cognitive impairment were excluded. The study protocol was approved by the Ethics Committee of the Beijing Anding Hospital, China.

### Instruments and evaluation

Patients’ and their carers’ demographic information was collected by two research psychiatrists in face-to-face interviews and was supplemented by a review of the electronic medical records. The HCL-33—Chinese version (Feng et al. 2016) was used to assess the patients' hypomanic symptoms. The Chinese version of the HCL-33 has been validated with good psychometric properties in both Chinese adults (Fang et al. 2019) and adolescents (Zhang et al. 2021). Patients’ carers completed the HCL-33-EA-Chinese version. Each item of the two scales has the dichotomous response format (yes/no). The total scores of the two HCL scales are calculated by adding up items with a “yes” response.

### Statistical analysis

All analyses were performed with the Statistical Package for Social Sciences (SPSS), Version 24.0. Patients’ and carers’ sociodemographic and clinical characteristics were compared between the BD and MDD groups; categorical variables were compared using chi-square tests, while normally distributed continuous variables were compared with two independent sample *t* tests; otherwise, Mann–Whitney U tests and Wilcoxon tests were used. Normality was examined with the one-sample Kolmogorov–Smirnov test. In order to explore the threshold for discriminating BD from MDD, receiver-operator curves (ROC) were generated and cut-off values were selected based on the Youden’s index from the respective curve (Youden 1950). The criterion validity of the HCL-33 and HCL-33-EA was estimated by sensitivity, specificity, PPV, NPV, and AUC. The association between the HCL-33 and the HCL-33-EA assessments was examined with Spearman correlation analysis. The level of statistical significance was set as P < 0.05 (two-tailed).

## Results

Of the 274 potential participants consecutively screened for the study, 260 met the inclusion criteria and completed the assessment;113 with MDD and 147 with BD. Table 1 presents the patients’ sociodemographic and clinical information; their mean age was 15.42 (standard deviation (SD) = 1.62) years; 22.69% were male. There was no significant difference of the demographic and clinical features between the MDD and BD groups. The vast majority of carers (93.85%) were married, 22.31% were male, and 12.69% were unemployed; their mean age was 43.10 (SD = 5.28) years, and their mean years in education was 13.17 (SD = 3.02) years (Table 2).

**Table 1:** Basic demographic and clinical characteristics of the study sample

Variables	Whole sample (n = 260)		MDD (n = 113)		BD (n = 147)		BD vs MDD	
	N	%	N	%	N	%	*χ*^2^	*P*
Male gender	59	22.69	31	27.43	28	19.05	2.561	0.110

	Mean	SD	Mean	SD	Mean	SD	Z^*^	*P*
Age (years)	15.42	1.62	15.30	1.51	15.51	1.70	− 1.015	0.310
Education level (years)	9.76	1.94	9.45	1.93	10.00	1.93	− 1.848	0.065
Age of onset (years)	14.33	4.21	14.21	5.40	14.41	3.01	− 1.170	0.242
Number of episodes	1.20	0.85	1.24	1.18	1.17	0.46	− 0.294	0.768

*BD* bipolar disorder, *MDD* major depressive disorder, *SD* standard deviation

^*^Mann–Whitney U test

**Table 2:** Demographic information of participants’ carers

Variables	Whole sample (n = 260)		Carers of MDD patients (n = 113)		Carers of BD patients (n = 147)		Carers for BD vs carers for MDD patients	
	N	%	N	%	N	%	*χ*^2^	*P*
Male gender	58	22.31	27	23.89	31	21.09	0.290	0.590
Unemployed	33	12.69	11	9.73	22	14.97	1.578	0.209
Married	244	93.85	108	95.58	136	92.52	1.035	0.309

	Mean	SD	Mean	SD	Mean	SD	Z^*^	*P*
Age (years)	43.10	5.28	43.09	6.42	43.10	4.22	–	0.348
Education level (years)	13.71	3.02	14.02	3.14	13.47	2.91	–	0.062

*BD* Bipolar Disorder, *MDD *Major Depressive Disorder, *SD* standard deviation

^*^Mann–Whitney U test

**Fig. 1:**
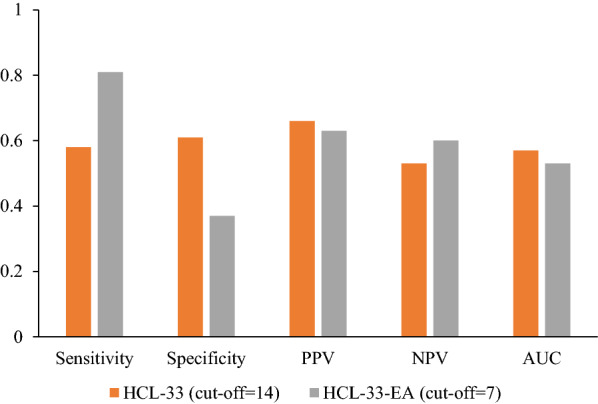
Sensitivity, specificity, positive predictive value (PPV), negative predictive value (NPV), and area under the curve (AUC) for the Hypomania Checklist-33 (HCL-33) and Hypomania Checklist-33 external assessment (HCL-33-EA) for Bipolar Disorder versus Major Depressive Disorder in adolescents

The mean scores of the HCL-33 and the HCL-33-EA were 13.38 (SD = 6.06) and 10.37 (SD = 5.52), respectively. The HCL-33 total score was significantly higher than that of the HCL-33-EA (Wilcoxon test; *Z* = − 6.478, *P* < 0.001) and the two scales’ scores were significantly and positively correlated (Spearman correlation coefficient: *rs* = 0.309, *P* < 0.001). The frequency of the items’ positive responses is shown in Table 3. Table 4 compares the sensitivity, specificity, PPV, NPV, and AUC between the HCL-33 and the HCL-33-EA in differentiating BD from MDD using cut-off values calculated according to Youden’ s index. The HCL-33 had a higher specificity and PPV, while the HCL-33-EA had a higher sensitivity and NPV (Table 3 and Fig. 1).

**Table 3:** Percentage of positive responses by adolescents with Major Depressive Disorder and Bipolar Disorder on the HCL-33 and HCL-33-EA

Items	Percentage of positive responses (%)			
	HCL-33		HCL-33-EA^ *^	
	BD	MDD	BD	MDD
1. I need less sleep	51.7	42.5	36.1	23.0
2. I feel more energetic and more active	64.6	57.5	66.7	52.2
3. I am more self-confident	41.5	39.8	52.4	43.4
4. I enjoy my work more	48.3	38.1	36.7	32.7
5. I am more sociable (make more phone calls, go out more)	53.7	41.6	38.8	38.1
6. I want to travel and/or do travel more	44.9	48.7	44.9	41.6
7. I tend to drive faster or take more risks when driving	27.2	17.7	8.8	8.0
8. I spend more money/too much money	56.5	46.9	37.4	23.9
9. I take more risks in my daily life (in my work and/or other activities)	32.7	29.2	11.6	8.0
10. I am physically more active (sport etc.)	39.5	30.1	27.9	15.9
11. I plan more activities or projects	57.1	42.5	47.6	32.7
12. I have more ideas, I am more creative	53.7	46.9	51.7	45.1
13. I am less shy or inhibited	40.8	28.3	38.1	24.8
14. I wear more colourful and more extravagant clothes/make-up	28.6	19.5	22.4	15.9
15. I want to meet or actually do meet more people	40.8	33.6	22.4	18.6
16. I am more interested in sex and/or I am more sexually active	19.7	11.5	9.5	5.3
17. I talk more	66.7	61.1	61.2	53.1
18. I think faster	55.1	54.9	61.2	54.0
19. I make more jokes or puns when I am talking	60.5	67.3	56.5	45.1
20. I am more easily distracted	53.1	54.9	36.7	40.7
21. I engage in lots of new things	51.7	41.6	42.9	35.4
22. My thoughts jump from topic to topic	68.0	65.5	32.0	30.1
23. I do things more quickly and/or more easily	45.6	46.0	49.0	37.2
24. I am more impatient and/or get irritable more easily	66.0	52.2	53.7	51.3
25. I can be exhausting or irritating for others	49.0	38.9	37.4	36.3
26. I get into more quarrels	43.5	35.4	27.2	23.9
27. My mood is higher, more optimistic	46.3	47.8	51.0	41.6
28. I drink more coffee	15.6	8.8	4.8	8.0
29. I smoke more cigarettes	8.2	2.7	2.7	2.7
30. I drink more alcohol	13.6	2.7	5.4	0.9
31. I take more drugs (sedatives, anxiolytics, stimulants…)	15.0	7.1	5.4	4.4
32. I game or gamble more	29.9	20.4	22.4	15.0
33. I eat more or I binge more	42.2	34.5	21.8	15.0

*BD* Bipolar Disorder, *MDD* Major Depressive Disorder, *HCL-33* 33-item Hypomania Checklist (HCL-33), *HCL-33-EA* 33-item Hypomania Checklist (HCL-33) external assessment

^*^The subject for each question of the HCL-33-EA is “He/She”

**Table 4:** Sensitivity, specificity, PPV, NPV, and AUC for the HCL-33 and HCL-33-EA in adolescents with Major Depressive Disorder and Bipolar Disorder

Scales	Cut-off value^*^	Sensitivity	Specificity	PPV	NPV	AUC	95% CI
HCL-33	14	0.58	0.61	0.66	0.53	0.57	0.48–0.65
HCL-33-EA	7	0.81	0.37	0.63	0.60	0.53	0.45–0.61

*95% CI* 95% confidence interval for AUC, *AUC* area under the curve, *BD*  bipolar disease, *HCL-33* 33-item Hypomania Checklist (HCL-33), *HCL-33-EA* 33-item Hypomania Checklist (HCL-33) external assessment, *MDD* Major Depressive Disorder, *NPV* negative predictive value, *PPV* positive predictive value

^*^The cut-off values were selected by Youden index from present study

## Discussion

An insufficient recognition of hypomanic symptoms by clinicians and by patients’ families leads to a failure to diagnose BD and subsequently to delayed or inappropriate treatment (Fagiolini et al. 2013). Although structured diagnostic interviews and screening instruments are available for identifying hypomania, to our knowledge, the HCL-33-EA is the only tool allowing patients’ carers to rate patients (Fang et al. 2019). The HCL-33-EA is user-friendly because it takes no more than fifteen minutes to administer and through the carers’ informed insight into the patient’s mood and behavior it can facilitate the early identification of hypomanic symptoms (Fang et al. 2019).

To the best of our knowledge, this was the first study that compared the psychometric properties of the self- and external assessment versions of the HCL-33 in adolescents. A significant and positive association between the total scores of the HCL-33 and the HCL-33-EA was found, similar to findings of the comparison between the HCL-33 and the HCL-33-EA in adult patients (Fang et al. 2019). More specifically, the mean score of the HCL-33-EA in this adolescent sample (10.37) was close to what was found in adults (11.0) (Feng et al. 2016). The total score of the HCL-33-EA was significantly lower than that of the HCL-33 in this study, which is also consistent with previous findings (Fang et al. 2019). In addition, compared to the HCL-33 self-assessment, external assessment with the HCL-33-EA had a higher sensitivity and negative predictive value, and a lower specificity and positive predictive value. This finding was consistent with that of a study comparing the HCL-33 and HCL-33-EA in an adult sample (Wang et al. 2021). Whilst the satisfactory sensitivity indicates that the HCL-33-EA may be an effective tool for differentiating BD from MDD, the lower specificity of HCL-33-EA may be the result of false positives. The discrepancy between the psychometric properties of the HCL-33 and HCL-33-EA in this study may be attributed to their different cut-off values. In a validation study in Russian adults (Mosolov et al. 2021), the HCL-33 cut-off value was 16, which is higher than the corresponding figure in this study. This discrepancy may be due to different target populations, i.e., adolescents vs. adults. A previous validation study on the HCL-33 in Chinese adolescents (Zhang et al. 2021) proposed a cut-off value of 18, which is higher than the cut-off value of 14 in this study. The discrepancy between the two studies is possibly due to different clinical characteristics of the participants. Similarly, the psychometric properties of the HCL-33-EA were less robust (specificity = 0.37), indicating that the HCL-33-EA needs further refinement for adolescent patients.

There were several limitations that need to be addressed. First, the study was conducted in a single center, which limits the generalizability of the findings. Second, due to the relatively small sample size, the two scales’ psychometric properties could not be compared separately by basic demographic variables, such as gender and age. Third, the ICD-10 is the official classification manual in clinical practice in China, therefore, we cannot identify bipolar subtypes I and II and compare psychometric properties between the HCL-33 and HCL-33-EA in distinguishing different BD subtypes from MDD. Fourth, information on psychiatric comorbidities is lacking in the electronic medical record system, preventing exploration of potentially confounding effects on the psychometric properties of the two instruments. Fifth, the adolescents included in this study were very young, therefore some of them diagnosed with MDD may be diagnosed with BD in the future, which may bias the findings of this study to an uncertain extent. Finally, although the consecutive sampling method was adopted, the proportion of girls was higher than expected, which may influence the findings of the study. However, the higher proportion of girls reflects the actual distribution of genders in clinical practice.

## Conclusion

In adolescents the sensitivity and negative predictive values of the HCL-33-EA were higher, while the specificity and positive predictive value were lower than the corresponding values in the HCL-33. In view of its high sensitivity, the HCL-33-EA would be more appropriate for screening for BD in depressed adolescents in Chinese clinical settings.

## Data Availability

The datasets generated and analyzed in the current study are not publicly accessible due to privacy and ethical restrictions.

## Abbreviations

AUC: Area under the curve
BD: Bipolar disorder
HAMD: Hamilton Depression Rating Scale
HCL-33: 33-Item Hypomania Checklist
HCL-33-EA: 33-Item Hypomania Checklist- external assessment
ICD-10: International Classification of Diseases, Tenth Revision
MDD: Major depressive disorder
MDQ: Mood Disorder Questionnaire
MINI: Mini-International Neuropsychiatric Interview
NPV: Negative predictive value
PPV: Positive predictive value
ROC: Receiver-operator curve
SCID-5: Structured Clinical Interview for DSM-5
SD: Standard deviation

## References

[CR1] Bae SO, Kim MD, Lee JG, Seo J-S, Won S-H, Woo YS (2014). Is it useful to use the Korean version of the mood disorder questionnaire for assessing bipolar spectrum disorder among Korean college students?. Asia Pac Psychiatry.

[CR2] Cuomo A, Aguglia A, Aguglia E, Bolognesi S, Goracci A, Maina G (2020). Mood spectrum symptoms during a major depressive episode: differences between 145 patients with bipolar disorder and 155 patients with major depressive disorder. Arguments for a dimensional approach. Bipolar Disords.

[CR3] Fagiolini A, Forgione R, Maccari M, Cuomo A, Morana B, Dell'Osso MC (2013). Prevalence, chronicity, burden and borders of bipolar disorder. J Affect Disord.

[CR4] Fang M, Wang YY, Feng Y, Ungvari GS, Ng CH, Wang G (2019). Exploration of the psychometric properties of the 33-item Hypomania Checklist - external assessment (HCL-33-EA). J Affect Disord.

[CR5] Feng Y, Xiang YT, Huang W, Wang G, Feng L, Tian TF (2016). The 33-item Hypomania Checklist (HCL-33): a new self-completed screening instrument for bipolar disorder. J Affect Disord.

[CR6] First M, Williams J, Karg R, Spitzer R (2015). Structured clinical interview for DSM-5—research version (SCID-5 for DSM-5, research version; SCID-5-RV).

[CR7] Hamilton M (1960). A rating scale for depression. J Neurol Neurosurg Psychiatry.

[CR8] Hong N, Bahk W-M, Yoon B-H, Shin YC, Min KJ, Jon D-I (2014). Characteristics of bipolar symptoms in psychiatric patients: pattern of responses to the Korean version of the Mood Disorder Questionnaire. Asia Pac Psychiatry.

[CR9] Hwang SHJ, Childers ME, Wang PW, Nam JY, Keller KL, Hill SJ (2010). Higher prevalence of bipolar I disorder among Asian and Latino compared to Caucasian patients receiving treatment. Asia Pac Psychiatry.

[CR10] Kriebel-Gasparro AM (2016). Advanced practice registered nurses: gateway to screening for bipolar disorder in primary care. Open Nurs J.

[CR11] Lecrubier Y, Sheehan DV, Weiller E, Amorim P, Bonora I, Sheehan KH (1997). The Mini International Neuropsychiatric Interview (MINI) A short diagnostic structured interview reliability and validity according to the CIDI. Eur Psychiatry.

[CR12] Łojko D, Dudek D, Angst J, Siwek M, Michalak M, Rybakowski J (2016). The 33-item Hypomania Checklist (HCL-33)—a study of the consistency between self- and external assessments in Polish bipolar patients. Psychiatr Pol.

[CR13] McIntyre RS, Calabrese JR (2019). Bipolar depression: the clinical characteristics and unmet needs of a complex disorder. Curr Med Res Opin.

[CR14] Mosolov SN, Yaltonskaya PA, Senko OV, Angst J (2021). Validation of the Russian version of the hypomania checklist (HCL-33) for the detection of bipolar disorder in patients with a current diagnosis of recurrent depression. J Affect Disord Rep.

[CR15] Nejati S, Ariai N, Björkelund C, Skoglund I, Petersson E-L, Augustsson P (2020). Correspondence between the neuropsychiatric interview M.I.N.I. and the BDI-II and MADRS-S self-rating instruments as diagnostic tools in primary care patients with depression. Int J Gen Med.

[CR16] Patella AM, Jansen K, Cardoso TA, Souza LDM, Silva RAD, Coelho F (2019). Clinical features of differential diagnosis between unipolar and bipolar depression in a drug-free sample of young adults. J Affect Disord.

[CR17] Phillips ML, Kupfer DJ (2013). Bipolar disorder diagnosis: challenges and future directions. Lancet.

[CR18] Wang Y-Y, Feng Y, Fang M, Guo C, Ungvari GS, Hall BJ (2021). Comparing screening abilities of the 33-item hypomania checklist (HCL-33) and the 33-item hypomania checklist external assessment (HCL-33-EA) for the detection of bipolar disorder. Front Psychiatry.

[CR19] World Health Organization (1992). The ICD-10 classification of mental and behavioural disorders: clinical descriptions and diagnostic guidelines.

[CR20] Xie G, Shen Q (1984). Use of the Chinese version of the Hamilton rating scale for depression in general population and patients with major depression. Chin J Nerv Ment Dis.

[CR21] Youden WJ (1950). Index for rating diagnostic tests. Cancer.

[CR22] Zhang Y, Li W, Zhang WY, He F, Pan HP, Cheung T (2021). Validation of the 33-item Hypomania Checklist (HCL-33) in screening adolescents with bipolar disorder. J Affect Disord.

[CR23] Zimmerman M, Galione JN (2011). Screening for bipolar disorder with the mood disorders questionnaire: a review. Harv Rev Psychiatry.

